# Downregulation of Glutathione-Mediated Detoxification Capacity by Binge Drinking Aggravates Acetaminophen-Induced Liver Injury through IRE1α ER Stress Signaling

**DOI:** 10.3390/antiox10121949

**Published:** 2021-12-05

**Authors:** Sou Hyun Kim, Hun Ji Choi, Hyeji Seo, Doyoung Kwon, Jaesuk Yun, Young-Suk Jung

**Affiliations:** 1 Department of Pharmacy, College of Pharmacy, Pusan National University, Busan 46241, Korea; souhyun@pusan.ac.kr (S.H.K.); oiooni@pusan.ac.kr (H.J.C.); hseo9393@pusan.ac.kr (H.S.); 2 Research Institute for Drug Development, Pusan National University, Busan 46241, Korea; 3 College of Pharmacy, Jeju National University, Jeju 63243, Korea; kwondoy@jejunu.ac.kr; 4 College of Pharmacy, Chungbuk National University, Cheongju 28160, Korea

**Keywords:** acetaminophen, alcohol, liver injury, glutathione, endoplasmic reticulum stress

## Abstract

Overdose of acetaminophen (APAP) can cause severe liver injury. Although alcohol is considered a risk factor for APAP toxicity, the mechanism underlying the interaction between alcohol and APAP remains unclear. Binge alcohol (5 g/kg every 12 h, 3 doses) reduced the concentration of cysteine and glutathione (GSH) and decreased expression of cystathionine β-synthase (CβS), cystathionine γ-lyase (CγL), and glutamate cysteine ligase catalytic subunit (GCLC) in the livers of male C57BL/6 mice. Furthermore, the levels of GSH S-transferase (GST) and GSH peroxidase (GPx) were decreased. To evaluate the effect of binge drinking on APAP-induced liver injury, 300 mg APAP was administered following alcohol binges. APAP in the binge group significantly amplified the serum ALT more than two fold and enhanced the pro-apoptotic proteins with a severe centrilobular necrosis compared to APAP alone. APAP treatment after alcohol binges caused lower levels of hepatic cysteine and GSH than APAP alone over 24 h, indicating that alcohol binges reduced GSH regenerating potential. Exposure to APAP after binge treatment significantly increased oxidative stress (lipid peroxidation) and endoplasmic reticulum (ER) stress (Grp78 and ATF6) markers at 6 h after treatment. Notably, the IRE1α/ASK1/MKK4/JNK pathway was activated, whereas CHOP expression was reduced by APAP administration in mice with pre-exposed alcohol binges compared with APAP alone. Thus, pretreatment with binge alcohol decreases GSH-mediated antioxidant capacity and contributes to augmentation of liver injury caused by subsequent APAP administration through differential ER stress signaling pathway.

## 1. Introduction

Acetaminophen (APAP), a commonly used analgesic and antipyretic drug, is safe at therapeutic doses, but it can cause severe liver injury in animals and humans by overdose administration [1]. APAP-induced hepatotoxicity accounts for half of the overdose-related acute liver failure in the United States [2]. When APAP is metabolized in proper doses, approximately 85–90% is removed from the urine via glucuronidation and sulfation. Approximately 5–10% of APAP is metabolized by CYP2E1 to form the toxic metabolite *N*-acetyl-p-benzoquinone imine (NAPQI) [3]. NAPQI is detoxified by conjugation with glutathione (GSH), an endogenous antioxidant, and is subsequently converted to mercapturic acid to release it out of the body. When the generation of NAPQI exceeds the availability of GSH for the conjugation reaction, the covalent adduct formation of NAPQI with macromolecules can lead to events correlating with the induction of necrosis in the liver [4].

Habitual and occasional excessive use of alcohol, known as binge drinking, are the two main risk behaviors related to alcohol consumption. Recent data have shown that binge drinking among adolescents is a risk factor for the development of alcohol-induced physical and mental disorders [5]. Although binge drinking itself causes a wide range of health problems, only a handful of medications have been demonstrated to have increased hepatotoxic potential in the setting of excessive alcohol use [6]. Alcohol is mainly metabolized in the liver by alcohol dehydrogenase (ADH) and aldehyde dehydrogenase (ALDH) to acetaldehyde and acetate, respectively. Approximately 10% of alcohol is metabolized by cytochrome P450 (CYP) 2E1, which has the highest activity for the production of reactive oxygen species (ROS) and acetaldehyde to initiate lipid peroxidation [7,8,9]. Since the Km value of CYP2E1 is much higher than that of ADH, metabolism by ADH is important when consumed with low concentrations of alcohol. However, high levels of alcohol saturate ADH and increase the metabolic flow mediated by CYP2E1. Chronic or binge alcohol administration induces the protein level of CYP2E1 to compensate for degradation by the macromolecular proteasome complex [10]. Induction of CYP2E1 by active alcohol consumption increases the biotransformation of certain medications that are also substrates for CYP2E1, which decreases their half-life and effectiveness. From a toxicological perspective, CYP2E1 activates many chemicals such as APAP, carbon tetrachloride, nitrosamine, acrylonitrile, and benzene to generate toxic intermediates [11,12,13,14,15]. Thus, it could be expected that the toxicity of these xenobiotics is enhanced in alcoholics.

It is considered that the induction of CYP2E1 and GSH depletion by alcohol consumption is directly attributable to the toxic effects of APAP [7,16]. In contrast, there are results in which APAP toxicity is suppressed by acute alcohol administration. In these studies, competitive inhibition of CYP2E1 by alcohol reduced the metabolism of APAP and inhibited excessive NAPQI production, eventually inhibiting APAP toxicity [17,18,19]. Although the risk of APAP toxicity associated with alcohol drinking has been known since the 1980s [20,21,22,23,24], it provides limited results to reflect the phenomenon and is not well defined for the mechanism study. In the present study, we investigated the effect of pretreated binge alcohol on APAP-induced liver injury by evaluating changes in GSH-related metabolic pathways reflecting detoxification capacity as well as endoplasmic reticulum stress signaling involved in cell death.

## 2. Materials and Methods

### 2.1. Animal and Treatments

Male C57BL/6 mice (7-weeks-old, *n* = 50) were purchased from Hyochang (Daegu, Korea). The mice were cared for according to the guidelines established and authorized by the Institutional Animal Care and Use Committee of Pusan National University (approval no. PNU-2021-2916). The mice were housed in a specific animal facility at standard room temperature (22 ± 2 °C), humidity (55 ± 5%) and a 12 h light and dark cycle. For one week of acclimatization to the experiment, the mice were orally administered three consecutive doses of alcohol (ethanol, 5 g/kg) or distilled water as vehicle at 12 h intervals [25]. APAP (300 mg/kg) was orally administered to mice 12 h after the last alcohol administration, designated as APAP 0 h [26]. The mice were sacrificed at 0, 2, 6, and 24 h after APAP treatment (Figure S1).

### 2.2. Examination of Serum Biochemical Parameters

Blood samples were collected via abdominal vein and transferred into a BD Microtainer Blood Collection Tube (BD Life Sciences, Franklin Lakes, NJ, USA). To obtain serum, the samples were centrifuged at 1300× *g* rpm for 20 min at 4 °C. Serum activities of alanine aminotransferase (ALT) were examined following the protocol of Reitman and Frankel [27]. ALT activity is equal to the quantity of pyruvate and oxaloacetate generated over time and are measured via a reaction with 2,4-Dinitrophenylhydrazine (DNPH) in alkaline solution. The absorbance was measured colorimetrically at 505 nm using a MULTISKAN GO reader (Thermo Scientific, Waltham, MA, USA).

### 2.3. Examination of Histological Mouse Liver Tissue

Liver tissues were fixed in 10% neutral-buffered formalin solution and embedded in paraffin for hematoxylin and eosin (H&E) staining. The tissues were sectioned (5 μm), mounted on glass slides, and examined under a light microscope (Olympus CX41RF, Olympus Co., Tokyo, Japan).

### 2.4. Examination of Hepatic Sulfur-Containing Metabolites

Liver lysate was obtained by homogenization of liver tissue with four-fold volume of buffer (150 mM NaCl, pH 7.4). The liver lysate was mixed with 1 M perchloric acid containing 2 mM EDTA or methanol, and the centrifuged (10,000× *g*, 10 min, 4 °C) supernatant was used for the determination of SAM/SAH/cysteine/GSH or methionine/taurine, respectively. The levels of cysteine and GSH were determined using HPLC with a fluorescence detector (excitation at 385 nm and emission at 515 nm; FLD-3100, Thermo Scientific). They were separated with a Hector M-C18 column (3 µm × 4.6 mm × 150 mm; Chiral Technology Korea, Daejeon, Korea) and fluorescence detector using the SBD-F derivation method. HPLC with Kromasil column (3 µm × 4.6 mm × 250 mm: Kromasil, Bohus, Sweden) and 254 nm UV detector (UltiMate™ 3000 VWD; Thermo Scientific) was used to analyze hepatic SAM and SAH concentration. Methione and taurine were derivatized using *o*-phthalaldehyde/2-mercaptoethanol and measured using an HPLC system with a fluorescence detector. They were separated using a Hector T-C18 column (3 µm × 4.6 mm × 100 mm; Chiral Technology Korea, Daejeon, Korea).

### 2.5. Examination of Hepatic Lipid Peroxidation

Hepatic lipid peroxidation was determined by measuring malondialdehyde (MDA) in the thiobarbituric acid reactive substances assay, as previously described [28]. The liver lysate was mixed with 6.7% trichloroacetic acid (TCA) for 15 min on ice and centrifuged at 10,000× *g* for 15 min at 4 °C. The supernatant was mixed with an equal volume of 0.67% thiobarbituric acid (TBA). Then, the mixture was incubated at 100 °C for 10 min. The complex levels of lipid peroxidation and TBA were measured colorimetrically at 532 nm using a MULTISKAN GO reader (Thermo Scientific).

### 2.6. Measurement of Hepatic ROS Generation

ROS generation was measured using 2′,7′-dichlorodihydrofluorescein diacetate (DCFDA), which was oxidized to highly fluorescent 2′,7′-dichlorofluorescein (DCF) by intracellular esterase and reactive species (ROS). To measure ROS levels, 10 μL of liver homogenates were added to 240 μL of 50 mM potassium phosphate buffer and 25 μM DCFDA (Molecular Probes, Eugene, OR, USA). A Glomax fluorescence plate reader (Promega, Madison, WI, USA) was used to monitor and quantify fluorescence intensity every 5 min for a total of 30 min with excitation and emission wavelengths of 485 and 535 nm, respectively.

### 2.7. Immunoblotting Analysis

The liver tissue was homogenized with ice-cold ProEX™ CETi protein extract solution (TransLab Biosciences, Daejeon, Korea) containing a protease and phosphatase inhibitor cocktail. The same amounts of protein were denatured and loaded onto sodium dodecyl sulfate-polyacrylamide gel electrophoresis (SDS-PAGE) and then transferred to nitrocellulose (NC) membranes (Bio-Rad, Hercules, CA, USA). The membranes were blocked with 5% skim milk for 30 min at 25 °C. They were then washed with Tris-buffered saline containing 0.1% Tween-20 (TBS-T) buffer. The following specific primary antibodies were incubated overnight at 4 °C (dilution 1:2000 to 1:5000). Anti-MAT1α, anti-CβS, anti-CγL, anti-GCLC (Santa Cruz Biotechnology, Santa Cruz, CA, USA), and anti- CDO (Abcam, Cambridge, MA, USA) antibodies were used for the detection of proteins involved in sulfur-containing amino acid metabolism. Anti-CYP2E1, anti-GST-α, anti-GST-μ and anti-GST-π (Detroit R&D, Detroit, MI, USA), anti-GPx, and anti-GR (Santa Cruz Biotechnology) antibodies were used for the determination of proteins for APAP metabolic activation and detoxification. Anti-4-HNE (Cell Signaling Technology, Danvers, MA, USA) and anti-nitrotyrosine (Santa Cruz Biotechnology) antibodies were used for examining lipid peroxidation and protein nitration resulting from oxidative and nitrosative stress, respectively. To detect proteins related with ER stress, anti-ATF6, anti-CHOP, anti-PERK (Santa Cruz Biotechnology), anti-Grp78, anti-IRE1α, and anti-p-IRE1α (Cell Signaling Technology) were used. To investigate the association of ER stress and apoptosis anti-p-MKK4, anti-JNK, anti-p-JNK (Cell Signaling Technology), and anti-p-ASK (Bioss antibody Inc., Woburn, MA, USA) antibodies were used to detect respective proteins. Anti-Bax, anti-Bcl-2, anti-cytochrome C, anti-caspase3, anti-cleaved caspase3, anti-PARP, and anti-cleaved PARP (Cell Signaling Technology) were used to examine apoptotic signaling pathway. The protein loading control, GAPDH, was detected using anti-GAPDH (Santa Cruz Biotechnology) antibody. The membrane was washed with TBS-T and incubated with the appropriate horseradish peroxidase-conjugated secondary antibody. The resulting antigen–antibody complexes were detected using the EZ-Western Lumi Pico detection kit (DOGEN, Seoul, Korea).

### 2.8. Statistical Analysis

All results are indicated as mean ± standard deviation (SD) and analyzed using two-tailed unpaired Student’s *t* test by using GraphPad Prism version 5.0 software (GraphPad Software, San Diego, CA, USA). The acceptable significance level was set at *p* < 0.05.

## 3. Results

### 3.1. Pretreatment with Alcohol Binges Potentiates APAP-Induced Hepatotoxicity in Mice

**Figure 1:**
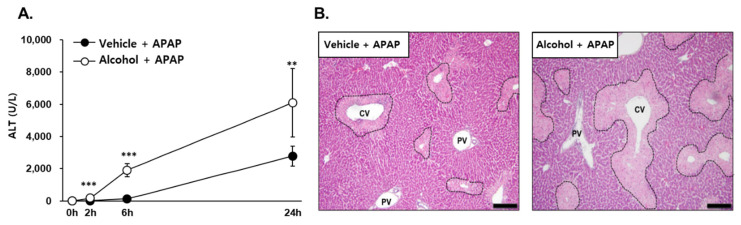
Alcohol pretreatment potentiates acetaminophen (APAP)-induced liver injury. Three consecutive doses of alcohol (5 g/kg, *n* = 20) or vehicle (*n* = 20) were administered orally to mice at 12 h intervals. Twelve hours later, following the last alcohol dose (designated as APAP 0 h), APAP (300 mg/kg) was administered orally to mice. (**A**) Time-dependent activity of ALT in serum was measured at 0 h (*n* = 5 in each group), 2 h (*n* = 5 in each group), 6 h (*n* = 5 in each group), and 24 h (*n* = 5 in each group). (**B**) Histopathology of hematoxyline and eosin (H&E)-stained liver tissues at 24 h after APAP treatment. The results are expressed as the mean ± SD. **, *** Significantly different from the vehicle + APAP group (Student’s *t* test, *p* < 0.05, *p* < 0.01 and *p* < 0.001, respectively). Scale bar, 200 μm; black dotted line, damaged area; CV, central vein; PV, portal vein.

Pretreatment of alcohol binges resulted in significantly enhanced liver injury by subsequent administration of APAP. An increase in serum ALT activity, a hepatotoxic parameter, of the pre-binge group compared to the vehicle group was evident from 2 h to 24 h after APAP treatment (Figure 1A). A histological analysis by H&E staining for examination of liver injury found that the alcohol + APAP group had a considerably increased severe and wide area of cetrilobular necrosis (Figure 1B).

### 3.2. Effects of Alcohol Binges on Hepatic Cysteine and GSH Concentration in Mice

**Figure 2:**
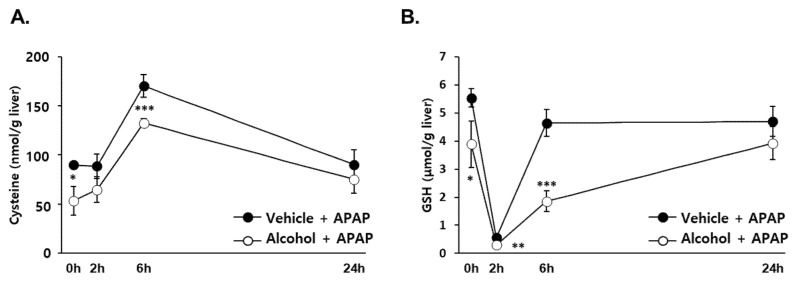
APAP pretreatment with alcohol changed cysteine, GSH concentration in the liver of mice. Three consecutive doses of alcohol (5 g/kg, *n* = 20) or vehicle (*n* = 20) were administered orally to mice at 12 h intervals. Twelve hours later, following the last alcohol dose (designated as APAP 0 h), APAP (300 mg/kg) was administered orally to mice. The mice were euthanized at 0 h (*n* = 5 in each group), 2 h (*n* = 5 in each group), 6 h (*n* = 5 in each group), and 24 h (*n* = 5 in each group) after APAP administration. Time-dependent levels of (**A**) hepatic cysteine and (**B**) GSH. The results are expressed as the mean ± SD. *, **, *** Significantly different from the vehicle + APAP groups (Student’s *t* test, *p* < 0.05, *p* < 0.01 and *p* < 0.001, respectively).

GSH synthesis is mainly regulated by the thiol-containing precursor cysteine, which provides GSH with the ability to maintain sulfhydryl homeostasis [29]. Because GSH is required for clearance of NAPQI through a conjugation reaction, its level is a critical factor in determining the extent of APAP-induced liver [4]. We monitored the hepatic concentrations of cysteine and GSH for 24 h after APAP administration to determine whether there is the differential effect of pretreated alcohol binges. The cysteine level in both groups increased to the maximum at 6 h after APAP administration and returned to the initial level at 24 h. Notably, alcohol + APAP showed a significantly lower concentration than that of vehicle + APAP at the time of maximal increase (Figure 2A). Hepatic GSH level was almost completely depleted 2 h after APAP administration. However, vehicle + APAP recovered within 6 h, whereas alcohol + APAP was restored slowly and recovered to the initial concentration at 24 h (Figure 2B). These results suggest that the ability to restore depleted GSH was reduced by alcohol binges.

### 3.3. Effects of Alcohol Binges on Hepatic Sulfur Amino Acid Metabolism in Mice

**Figure 3:**
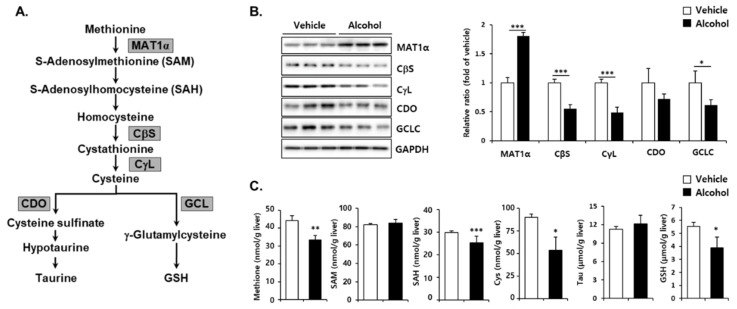
Alcohol changes the hepatic enzymes involved in trans-sulfuration pathway and sulfur-containing metabolites in the liver of mice. Three consecutive doses of alcohol (5 g/kg, *n* = 5) or vehicle (*n* = 5) were administered orally to mice at 12 h intervals. The mice were euthanized 12 h after the last alcohol treatment (designated as APAP 0 h). (**A**) Sulfur-containing amino acid metabolism. (**B**) Methionine adenosyltransferase (MAT1α), cystathionine β-synthase (CβS), cystathionine γ-lyase (CγL), cysteine dioxygenase (CDO) and glutamate cysteine ligase catalytic subunit (GCLC) protein levels were evaluated by immunoblotting in the liver at APAP 0 h. GAPDH was used as the loading control. The expression levels of proteins were normalized to that of GAPDH. (**C**) Hepatic concentrations of methionine, *S*-adenosylmethionine (SAM), *S*-adenosylhomocysteine (SAH), cysteine, taurine and glutathione (GSH) were measured in the liver at APAP 0 h. The results are expressed as the mean ± SD. *, **, *** Significantly different from the vehicle group (Student’s *t* test, *p* < 0.05, *p* < 0.01 and *p* < 0.001, respectively).

To further determine the effect of alcohol binges on the metabolic pathway for synthesis of liver GSH, changes of sulfur amino acids as well as corresponding enzymes (Figure 3A) were measured in vehicle- and alcohol-treated mice. Hepatic MAT1α protein levels were markedly increased, but CβS, CγL, and GCLC protein levels were significantly reduced by binge alcohol administration. CDO, mediating taurine synthesis, was not changed (Figure 3B). Hepatic methionine concentration was decreased and there was no significant alteration in SAM levels by alcohol intake (Figure 3C). Binge alcohol administration resulted in decreased SAH, cysteine, and GSH in the liver. The level of taurine was not changed. Cysteine is irreversibly metabolized in the liver to yield either taurine or GSH [30,31]. A decrease in liver GSH by alcohol binges appears to be associated, at least in part, with depression of GCLC, the rate-limiting enzyme for GSH synthesis, as well as cysteine availability for generation of taurine.

### 3.4. Alcohol Binges Induced Oxidative Stress in Mice Liver

**Figure 4:**
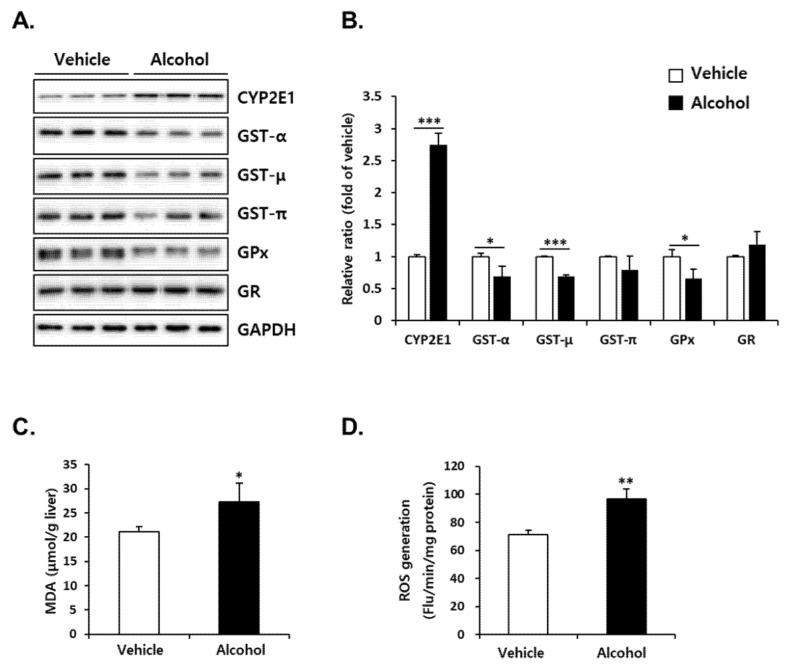
Alcohol binges changed hepatic GSH metabolism in mice. Three consecutive doses of alcohol (5 g/kg, *n* = 5) or vehicle (*n* = 5) were administered orally to mice at 12 h intervals. The mice were euthanized 12 h after the last alcohol treatment (designated as APAP 0 h). (**A**) The hepatic CYP2E1, glutathione S-transferase-alpha (GST-α), -mu (GST-μ), -pi (GST-π), glutathione peroxidase (GPx) and glutathione reductase (GR) protein levels were evaluated by immunoblotting in the liver at APAP 0 h. GAPDH was used as the loading control. (**B**) The expression levels of proteins were normalized to that of GAPDH. Only alcohol pre-treatment changes in the (**C**) hepatic malondialdehyde (MDA) and (**D**) hepatic reactive oxygen species (ROS) levels. *, **, *** Significantly different from the vehicle group (Student’s *t* test, *p* < 0.05, *p* < 0.01 and *p* < 0.001, respectively).

Reduced GSH levels implicate disruption of liver redox homeostasis. For identification of additional changes in the redox environment, we measured the protein expression of CYP2E1, which generates ROS during metabolism of alcohol, as well as GSH-related antioxidant enzymes. Alcohol administration increased hepatic CYP2E1 protein to 250% of vehicle at 12 h after the final dosing (Figure 4A). The protein levels of GSH-dependent antioxidant enzymes including GST-α, -μ, GPx in the liver of alcohol-treated mice were significantly lower than those of vehicle (Figure 4B). Binge alcohol did not induce a significant change in GST-π and GR (Figure 4B). Subsequent measurement of oxidative stress indicators such as MDA, a lipid peroxidation end product of polyunsaturated fatty acids (PUFAs), (Figure 4C) and ROS (Figure 4D) levels showed that alcohol binges induced significant oxidative stress in the liver.

### 3.5. Alcohol Binges Induced ER Stress in Mice Liver

**Figure 5:**
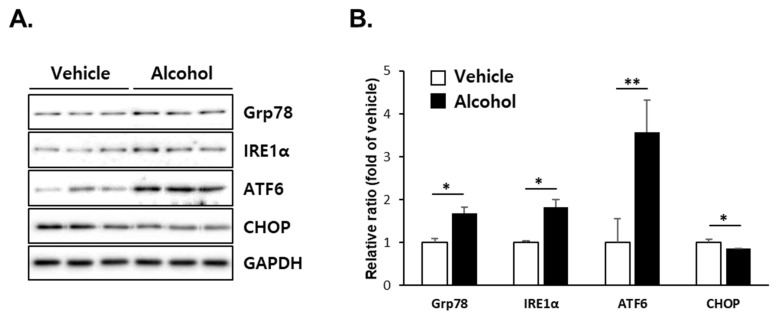
Alcohol binges increased hepatic ER stress in the liver of mice. Three consecutive doses of alcohol (5 g/kg, *n* = 5) or vehicle (*n* = 5) were administered orally to mice at 12 h intervals. The mice were euthanized 12 h after the last alcohol treatment (designated as APAP 0 h). (**A**) Immunoblot analysis showing the protein levels of glucose-regulated protein (Grp78), inositol-requiring enzyme 1α (IRE1α), activating transcription factor 6 (ATF6) and c/EBP homology protein (CHOP) in mice liver tissues at APAP 0 h. GAPDH was used as the loading control. (**B**) The expression levels of proteins were normalized to that of GAPDH. The results are expressed as the mean ± SD. *, ** Significantly different from the vehicle group (Student’s *t* test, *p* < 0.05, *p* < 0.01 and *p* < 0.001, respectively).

ER stress has been proposed as an important mechanism for the progression of alcoholic liver injury [32,33,34,35]. In particular, dysregulation of sulfur amino acid metabolism due to chronic alcohol intake is known as one of the risk factors for inducing ER stress [35,36,37]. In line with this, we sought to determine whether alcohol binges induce an ER stress response. Higher levels of Grp78, IRE1α, and ATF6 were observed in the alcohol-treated group compared with those of the vehicle-treated group, while the CHOP protein level was downregulated to 80% of the vehicle-treated group at 12 h after the final alcohol administration (Figure 5).

### 3.6. Pretreatment with Alcohol Binges Amplified APAP-Induced Oxidative Stress in Mice Liver

**Figure 6:**
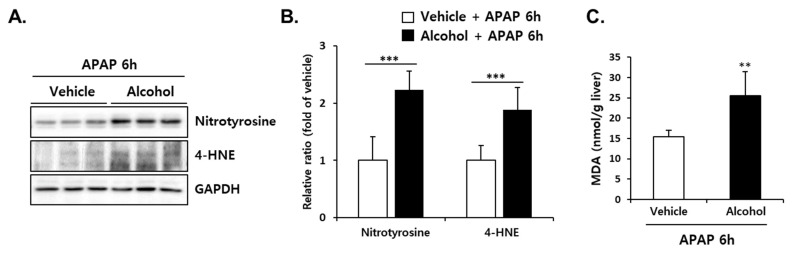
Alcohol binges enhanced oxidative stress in the liver of mice. Three consecutive doses of alcohol (5 g/kg, *n* = 5) or vehicle (*n* = 5) were administered orally to mice at 12 h intervals. Twelve hours later, following the last alcohol dose, APAP (300 mg/kg) was administered orally to mice. The mice were euthanized 6 h after APAP administration (APAP 6 h). (**A**) At 6 h after APAP administration, the levels of oxidized proteins of nitrotyrosine and 4-hydroxynonenal (4-HNE) were detected. GAPDH was used as the loading control. (**B**) The expression levels of proteins were normalized to that of GAPDH. (**C**) Hepatic malondialdehyde (MDA) levels were detected 6 h after APAP administration (APAP 6 h). The results are expressed as the mean ± SD. **, *** Significantly different from the vehicle + APAP 6 h group (Student’s *t* test, *p* < 0.01 and *p* < 0.001, respectively).

To investigate the effect of alcohol binges on APAP-induced oxidative stress, hepatic levels of MDA, nitrotyrosine-protein adducts, and 4-HNE, another lipid peroxidation product from PUFAs, were measured at 6 h after APAP administration with/without pre-treatment of alcohol binges. As shown in Figure 6A,B, alcohol + APAP significantly induced the levels of nitrotyrosine-protein adducts and 4-HNE compared with vehicle + APAP. Differences in liver MDA levels (Figure 6C) were consistent with changes in nitrotyrosine-protein adducts and 4-HNE levels, indicating that hepatic oxidative stress increased by alcohol binges was exacerbated by subsequent administration of APAP.

### 3.7. Administration of APAP in Mice with Pretreated Alcohol Binges Activates IRE1α/ASK1/MKK4/JNK Signal

**Figure 7:**
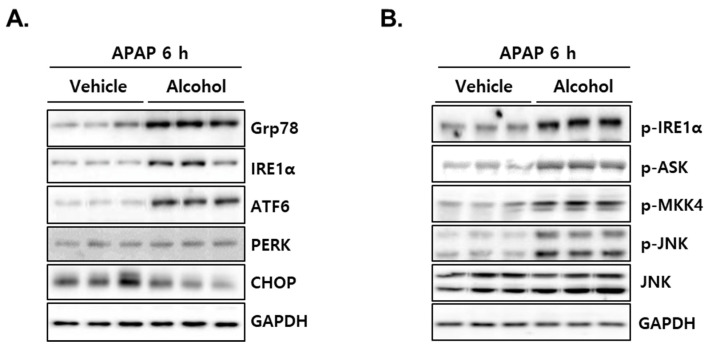
Alcohol binges enhanced IRE1α-mediated JNK activation. Three consecutive doses of alcohol (5 g/kg, *n* = 5) or vehicle (*n* = 5) were administered orally to mice at 12 h intervals. 12 h later, following the last alcohol dose, APAP (300 mg/kg) was administered orally to mice. The mice were euthanized 6 h after APAP administration (APAP 6 h). (**A**) Immunoblot analysis showing the protein levels of Grp78, IRE1α, ATF6, PERK and CHOP in mice liver tissues at APAP 6 h. (**B**) Immunoblot analysis showing the protein levels of p-IRE1α, p-ASK, p-MKK4, p-JNK and JNK in mice liver tissues at APAP 6 h. GAPDH was used as the loading control.

According to accumulated reports, CHOP activation via ER stress response is one of the important mechanisms in APAP-induced acute liver injury [38,39,40]. Therefore, we explored the ER stress-mediated cell death signaling pathway to elucidate the mechanism by which liver injury was amplified in the alcohol + APAP group. As shown in Figure 7A, alcohol + APAP showed significant induction of a crucial ER chaperone, Grp78, as well as ER-localized stress sensors such as IRE1α, ATF6. Another ER-localized stress sensor, PERK, did not change, and, unexpectedly, the expression of CHOP was decreased in the liver of alcohol + APAP mice (Figure 7A). In further investigation of ER stress-mediated cell signaling, alcohol + APAP showed significantly induced IRE1α-ASK-MKK4-JNK activity (Figure 7B). These results suggest that APAP administration after alcohol intake can amplify liver injury through a signaling pathway that is different from CHOP activation by APAP alone.

### 3.8. Pretreatment with Alcohol Binges Potentiates APAP-Induced Apoptosis in Mice Liver

**Figure 8:**
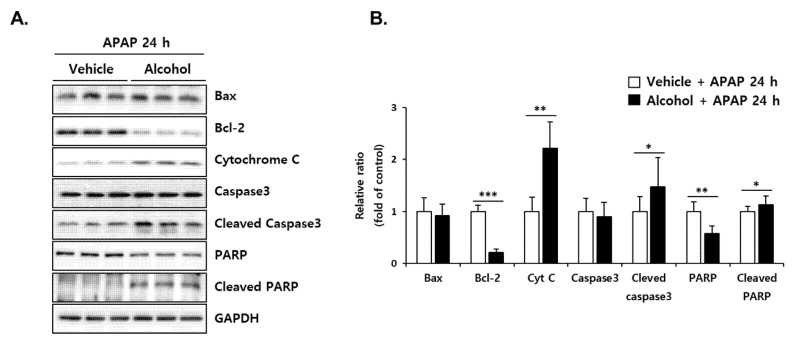
Alcohol binges enhanced ER stress-mediated apoptosis. Three consecutive doses of alcohol (5 g/kg, *n* = 5) or vehicle (*n* = 5) were administered orally to mice at 12 h intervals. Twelve hours later, following the last alcohol dose, APAP (300 mg/kg) was administered orally to mice. The mice were euthanized 24 h after APAP administration (APAP 24 h). (**A**) Immunoblot analysis showing the protein levels of Bax, Bcl-2, Cytochrome C, caspase3, Cleaved Caspase3, PARP, and Cleaved PARP in mice liver tissues at APAP 24 h. GAPDH was used as the loading control. (**B**) The expression levels of proteins were normalized to that of GAPDH. *, **, *** Significantly different from the vehicle + APAP 24 h group (Student’s *t* test, *p* < 0.05, *p* < 0.01 and *p* < 0.001, respectively).

Protein expression of the apoptotic signaling cascade was examined at 24 h post-APAP administration (Figure 8). Although Bax was not changed, the alcohol + APAP exhibited significantly higher expression of pro-apoptotic proteins (cytochrome C, cleaved caspase3, cleaved PARP) and a lower level of anti-apoptotic protein (Bcl-2) than vehicle + APAP. These results suggest that APAP administration in mice pre-exposed to alcohol binges enhances the apoptosis signal compared to the APAP alone group, which could eventually lead to amplification of liver injury.

## 4. Discussion

APAP is classified as a highly effective over-the-counter pain reliever, and therefore, one of the drugs that has been with us for a long time. Although many studies have been conducted on its side effects, there is still much to be done about whether lifestyle can increase the harmful effects of this medication. In particular, most people who consume alcohol also take medications, and it is known that many medications have the potential to interact with alcohol. These interactions can alter the metabolism or activity of drugs, sometimes resulting in serious medical consequences. In this study, alcohol binges weakened the antioxidant capacity in the liver through the suppression of GSH synthesis as well as GSH-mediated oxidant-removing enzymes. Subsequent exposure to APAP potentiated liver injury as compared to APAP alone. APAP pre-treatment with alcohol significantly activated the IRE1α/ASK1/MKK4/JNK signal, an ER stress-mediated cell death-inducing pathway, and it is considered to be an important mechanism amplifying liver injury, which is distinct from the APAP-only group.

It is well documented that alcohol administration reduces GSH levels in the liver, and various mechanisms have been proposed. Increased lipid peroxidation or acetaldehyde generation by alcohol treatment is considered a major cause of hepatic GSH depletion [41,42,43]. Based on kinetic studies, it has been suggested that alcohol increases GSH efflux from the liver and/or decreases biosynthesis, followed by a reduction in hepatic GSH levels [44,45,46]. It has also been suggested that acute alcohol administration not only inhibits cysteine synthesis but also enhances cysteine availability for taurine synthesis, which plays an important role in the depletion of hepatic GSH [47]. However, there are still limitations in understanding the physiological and pathological implications of GSH changes from the perspective of GSH-related metabolic pathways. To address the effect of binge drinking on GSH-mediated antioxidant capacity, we focused on the metabolic pathway to synthesize GSH and remove reactive substances using GSH. We found that alcohol binges repress the de novo synthesis of GSH due to a decrease in both the substrate cysteine level and the rate-limiting enzyme GCLC protein expression (Figure 3). Furthermore, the expression levels of GPx, which convert reactive H_2_O_2_ to H_2_O, and GSTs to remove reactive substances by conjugation reactions were decreased (Figure 4). This was accompanied by increased lipid peroxidation and ROS generation, suggesting that the redox imbalance caused by alcohol binge eventually caused oxidative stress. Thus, our findings indicate that alcohol binges weaken hepatic antioxidant capacity by downregulating GSH synthesis as well as GSH consumption to quench reactive substances.

GSH, a tripeptide composed of cysteine, glycine, and glutamate, plays a pivotal role in the removal of NAPQI from the oxidation of APAP. Both non-enzymatic and GST-mediated reactions are involved in the conjugation reaction, and in the presence of high concentrations of GSH and NAPQI, rapid non-enzymatic detoxification is dominant [48,49]. Because the concentration of glutamate and glycine is much higher than that of cysteine in the liver, the changes in cysteine levels are a critical determinant in the synthesis of GSH [30]. To investigate changes in cysteine availability to synthesize GSH after APAP treatment, we analyzed the hepatic concentration over 24 h. GSH depletion rapidly occurred and recovered in the liver of APAP-treated mice, whereas pre-treatment with binge alcohol prolonged APAP-induced GSH depletion and persisted at lower levels throughout the experimental period (Figure 2). Cysteine concentrations increased up to 6 h after APAP treatment, which may explain the enhanced utilization of this amino acid to replenish rapidly depleted GSH through biosynthesis (Figure 2). Moreover, lower levels of cysteine in alcohol binges compared with APAP alone, probably due to a decrease in its supply through the trans-sulfuration pathway. These results suggest that alcohol binges reduce the regenerating potential of GSH after consumption, followed by an enhancement of APAP toxicity in the liver.

ER is a specialized organelle responsible for cell homeostasis and survival through functions such as protein folding, lipid biosynthesis, and calcium and redox balance [50]. Specifically, redox homeostasis of the ER lumen is an important part of the oxidative folding of secretory proteins, and the oxidative environment resulting from changes in the ER redox status can interfere with the protein folding process, causing ER stress to remove misfolded and unfolded proteins [51]. We noted that there are most phase I enzymes including CYPs and some phase II enzymes in the ER, which are important for xenobiotic metabolism, including biotransformation of alcohol and APAP [52]. Thus, depletion of the intraluminal GSH pool in the ER by these chemicals results in the abrogation of redox homeostasis, and subsequent induction of ER stress may be inevitable [53]. In this study, binge drinking itself induced ER stress as well as oxidative stress accompanied by a decrease in GSH without obvious liver injury. In response to ER stress, the levels of ATF6 and IRE1α increased, whereas CHOP expression was decreased (Figure 5). These results suggest that the disturbance of liver homeostasis by binge drinking induced ER responses, which did not lead to cellular apoptotic signaling.

A number of studies have shown that ER stress response molecules are involved in the pathogenesis of APAP-induced liver injury. Specifically, treatment with APAP at hepatotoxic doses significantly induced the ER stress-responsive proapoptotic transcription factor CHOP [38,39], and APAP-induced liver injury was protected by CHOP deficiency [40]. These results suggest that CHOP is a critical mediator of APAP-induced hepatotoxicity. In contrast, the activation of IRE1α rescued liver injury by toxic dose administration of APAP [54]. In that study, constitutive activation of IRE1α by XBP1 ablation in the liver degraded mRNA of Cyp1a2 and Cyp2e1, key enzymes oxidizing APAP, which prevented APAP intoxication accompanied by reduced JNK activation. However, in their results, GSH depletion and APAP-protein adduct did not show significant differences between wild-type and XBP1-deficient mice. Thus, it is unclear whether inhibition of metabolic oxidation by IRE1α contributed to the protection of APAP intoxication. In our study, particularly noteworthy is the activation of IRE1α/ASK1/MKK4/JNK signaling, while CHOP expression was reduced by APAP administration in mice with pre-exposed alcohol binges (Figure 7). In addition to enodoribonuclease activity, IRE1α has a serine/threonine kinase domain and binds the adaptor protein, TNF receptor-associated factor-2 (TRAF2), which then promotes the activation of c-Jun N-terminal kinase (JNK) through apoptosis signal-regulating kinase-1 (ASK1) [55,56,57]. ASK1 is a member of the mitogen-activated protein (MAP) kinase kinase family, which activates the MKK4/MKK7-JNK MAPK pathway and constitutes a pivotal signaling pathway in various types of stress-induced apoptosis [58,59,60]. Thus, our findings suggest that IRE1α-mediated ER stress signaling could play a critical role in the potentiation of APAP-induced liver injury in pre-exposed binge alcohol. Since ROS also can activate ASK, it is still unclear whether ER stress was the major upstream signal of ASK-dependent apoptosis in our results, and thus the exact mechanism needs to be clarified in future studies.

## 5. Conclusions

In conclusion, repeated binge alcohol abrogated the hepatic antioxidant defense system by sustained reduction of GSH synthesis, as well as the ability to remove GSH-mediated reactive substances. Subsequent administration of APAP showed that pre-exposure to binge drinking was an important risk factor in increasing the extent of liver damage compared to APAP alone. The kinetic evaluation of cysteine and GSH concentrations after APAP treatment suggests that alcohol binges downregulate GSH-regenerating potential, accounting for the inefficient clearance of reactive metabolites of APAP. The differential response to APAP-induced ER stress in the liver of alcohol binges was identified as an activated IRE1α/ASK1/MKK4/JNK pathway, suggesting a distinct cell death signal that increases sensitivity to acute liver injury.

## Data Availability

The data presented in this study are available in this article.

## Supplementary Materials

The following are available online at https://www.mdpi.com/article/10.3390/antiox10121949/s1, Figure S1: Experimental schedule.
