# Supplementary figures and images for: Downregulation of Glutathione-Mediated Detoxification Capacity by Binge Drinking Aggravates Acetaminophen-Induced Liver Injury through IRE1α ER Stress Signaling
Source: Antioxidants (Basel). 2021 Dec 5;10(12):1949. doi: 10.3390/antiox10121949 (PMC8750905; doi:10.3390/antiox10121949)

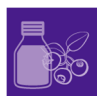

## Supplementary figure

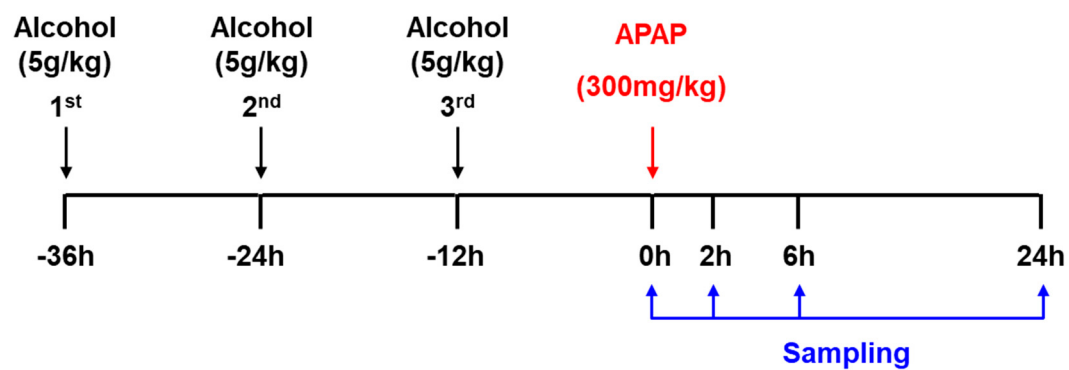

Figure S1. Experimental schedule

Supplement: Supplementary file 1 [file antioxidants-10-01949-s001.zip › antioxidants-1475475-supplementary.pdf]
